# Case Report: Comprehensive Imaging and Clinical Outcomes of Chondro-Osseous Respiratory Epithelial Adenomatoid Hamartoma (COREAH) in a Dog

**DOI:** 10.3390/ani15233389

**Published:** 2025-11-24

**Authors:** Won-Keun Park, Jun-Won Yoon, Chan-Sik Nam, Dong-Min Choi, Kwang-Sup Lee, Yeon-Jin Kim, Tae-Jung Dan, Dong-Hoon Shin, Hee-Myung Park

**Affiliations:** 1 Yonggang Animal Hospital, Daeheungro 38, Seoul 04162, Republic of Korea; goodvet@nate.com (W.-K.P.); neodhoon@gmail.com (D.-H.S.); 2 Laboratory of Veterinary Internal Medicine, College of Veterinary Medicine, Konkuk University, Neungdongro 120, Seoul 05029, Republic of Korea; dkrk9454@gmail.com (J.-W.Y.); enzmahsnam@naver.com (C.-S.N.); alsehdtmajvm@gmail.com (D.-M.C.); deu02181@naver.com (K.-S.L.); grateyj@naver.com (Y.-J.K.); dantae162534@naver.com (T.-J.D.)

**Keywords:** dog, respiratory epithelial adenomatoid hamartoma, chondro-osseous respiratory epithelial adenomatoid hamartoma, endoscopy, rhinoscopy

## Abstract

The aim of this case report is to describe comprehensive imaging such as CT scanning, endoscopy, histopathology, and clinical outcomes following medical and endoscopic treatment. A 5-year-old spayed female Chihuahua presented with chronic unilateral nasal discharge, congestion, and intermittent stertor, unresponsive to initial antibiotics and steroids. Further diagnostics, including endoscopy, revealed a cystic lesion with osseous fragments in the left nasal cavity, while CT imaging identified a soft tissue mass with associated bone resorption. Histopathology confirmed chondro-osseous respiratory epithelial adenomatoid hamartoma (COREAH), a rare benign lesion characterized by respiratory epithelium, fibrovascular proliferation, and osseous components, along with lymphoplasmacytic and eosinophilic inflammation. Clinical signs improved following endoscopic aspiration and drainage, which restored nasal cavity space. This case highlights the importance of combining endoscopy, advanced imaging, and histopathology for accurate diagnosis and effective management of COREAH, a condition with potential for recurrence. The use of endoscopy enabled real-time visualization and precise tissue sampling, distinguishing this case from others relying solely on imaging. This report contributes to the limited veterinary literature on COREAH and underscores the need for long-term monitoring due to its uncertain pathogenesis and variable presentation.

## Simple Summary

This report describes a rare type of non-cancerous growth found inside the nose of a small dog. The dog, a 5-year-old Chihuahua, had long-lasting symptoms such as nasal discharge and noisy breathing that did not get better with regular medication. A CT scan and endoscope showed a fluid-filled lump pushing on nearby tissue. A sample of the lump was taken and tested, confirming a rare condition called COREAH. This is a slow-growing lump made of normal tissue found in the nose, along with small pieces of bone and signs of inflammation. After using the endoscope to drain the fluid from the lump, the dog’s breathing and nasal discharge improved. This case shows how using both imaging and a camera exam helps find the cause of unusual nasal problems in pets. This report aims to raise clinical awareness of this rare entity and to assist clinicians in making accurate diagnostic and therapeutic decisions.

## 1. Introduction

Respiratory epithelial adenomatoid hamartoma (REAH) is a rare benign lesion that affects the upper respiratory tract, first described in humans in 1995. Clinically, REAH presents symptoms such as nasal discharge, facial pain, pressure, headaches, and impaired sense of smell [1]. Histologically, REAH is characterized by a polypoid structure with glandular adenomatoid proliferations lined by respiratory epithelium [2]. A rare subset of REAH, known as COREAH, contains a central core of chondro-osseous matrix [3]. COREAH is extremely rare, with only 18 human cases reported, including both adults and children [3,4].

Unlike human cases, canine COREAH often presents significant bone destruction, likely caused by compression atrophy rather than direct bone invasion, as benign hamartomas typically do not infiltrate bone tissue [1,5]. Diagnostic imaging, especially CT scans, in dogs typically reveals unilateral soft tissue masses with multifocal mineralization and bone loss, initially raising concern for a more aggressive neoplastic process [3,6]. However, its classification as a hamartoma, inflammatory lesion, or neoplasm remains uncertain due to its mixed histological features and potential for recurrence [5]. The pathogenesis of both REAH and COREAH is still controversial, with some theories suggesting a reactive inflammatory process, while others propose a neoplastic origin [1]. Surgical excision is the standard treatment for REAH and is generally curative, though COREAH may have a higher risk of recurrence [6,7]. In veterinary cases, treatment protocols are limited due to the rarity of COREAH. A dorsal rhinotomy with turbinectomy has been employed in some dogs to improve mass visualization and facilitate tissue removal and biopsy [3,6]. This case aims to describe comprehensive imaging such as CT scanning and endoscopy and histopathology, and clinical outcomes following medication and endoscopic treatment.

## 2. Case Description

A 5-year-old female spayed Chihuahua, weighing 4.98 kg, was admitted to the hospital for evaluation due to a history of nasal discharge, congestion, and stertor in the left nasal cavity. The patient initially visited a local clinic, presenting with clear, foamy nasal discharge on one side and stertor. The dog had steroid responses for 2 months and showed clinical improvement during treatment; however, the symptoms recurred after discontinuation. Cytological examination revealed the presence of bacteria, prompting antibiotic therapy. However, the clinical signs recurred during ongoing use of the medication.

**Figure 1:**
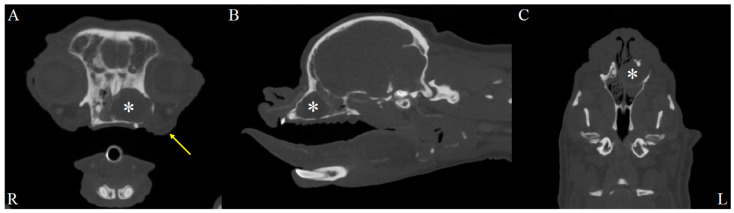
Soft tissue window, post-contrast CT images of the dog’s head. Axial (**A**), sagittal (**B**), and dorsal (**C**) post-contrast CT images in the soft tissue window reveal a lesion in the left nasal cavity (R: right, L: left). A fluid-attenuated structure (asterisks) measuring 1.35 cm in height, 1.68 cm in width, and 1.49 cm in length is observed in the mid-region of the left nasal cavity. Additionally, soft tissue swelling is evident in the left perinasal region (yellow arrow), suggesting involvement of adjacent soft tissues.

**Figure 2:**
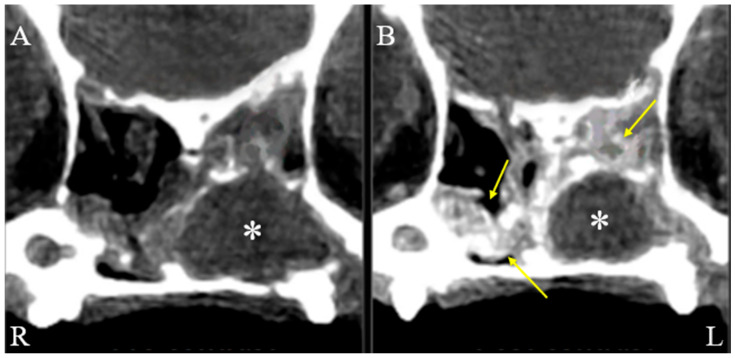
Axial, soft tissue window, pre (**A**) and post (**B**) contrast CT images of the dog’s nasal cavity. Axial soft tissue window CT images of a dog’s nasal cavity (R: right, L: left), comparing pre-contrast (**A**) and post-contrast (**B**) scans. Surrounding the mass (asterisks), soft tissue-attenuated structures with contrast enhancement (arrows) are visible within the nasal cavities, indicating soft tissue changes adjacent to the mass.

On physical examination, the dog showed serous nasal discharge in the left nasal cavity, nasal congestion, and intermittent reverse sneezing. Other physical examinations were unremarkable. Based on these findings, further computed tomographic (CT) evaluation of the nasal cavity was required. The dog was premedicated with butorphanol (0.2 mg/kg, intramuscular injection, Myungmoon, Seoul, Republic of Korea). Anesthesia was induced with propofol (6 mg/kg, intravenous injection, Myungmoon, Seoul, Republic of Korea). Intubation was performed, and anesthesia was maintained with isoflurane. CT (MyVet CT i3D, Woorien, Seoul, Republic of Korea) images of the nasal cavity were obtained. CT findings revealed a fluid-attenuated structure measuring 1.35 × 1.68 × 1.49 cm located in the mid-region of the left nasal cavity. The lesion involved the left nasal cavity, accompanied by soft tissue swelling in the adjacent left perinasal region, suggesting possible pathology in both the nasal cavity and surrounding soft tissues (Figure 1). The scan also revealed a fluid-attenuated mass occupying the nasal cavity, with evident contrast enhancement in the adjacent soft tissues, indicating pathological involvement of the surrounding structures (Figure 2).

**Figure 3:**
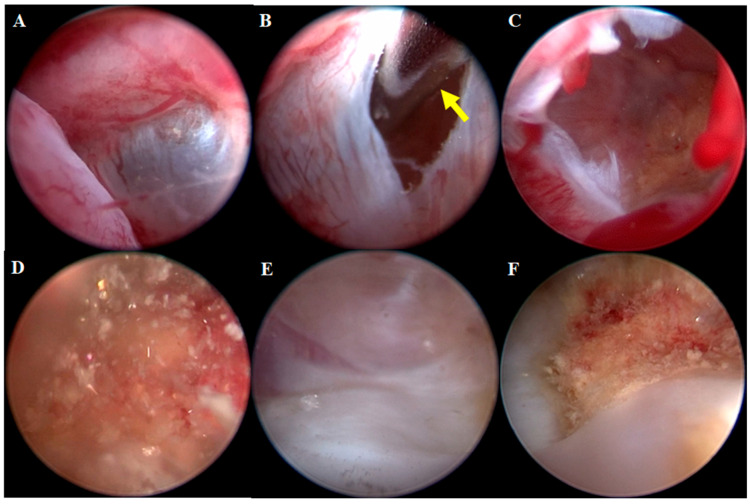
Endoscopic Images of the Cystic Lesion’s Interior and Exterior. Endoscopic image of the external appearance of the lesion (**A**). The cystic wall exhibits elastic consistency and is vascularized, with its semi-transparent nature allowing the internal contents to be visible through the wall. The wall was punctured with a myringotomy needle with yellow arrow (**B**). The interior is revealed, demonstrating a cavity structure filled with clear fluid (**C**). Floating cholesterol particles were observed inside the cyst (**D**). The ventral and caudal walls of the cyst display a low proportion of vascularized mucosa and are composed of smooth bone or cartilaginous tissue (**E**). A patchy lesion was noted on the floor of the cyst, and the particles observed within the cyst are likely derived from this patchy lesion (**F**).

Given the CT findings indicating a fluid-filled structure within the left nasal cavity and possible involvement of adjacent soft tissues, nasal endoscopy was subsequently performed to better characterize the lesion and collect biopsy specimens. An endoscopic examination was performed with the dog in sternal recumbency to examine the nasal cavity. Inspection of the nasopharynx and choanae was evaluated using retroflex rhinoscopy (Video Uretero-Renoscope FLEX-XC, 11278VSK Karl Storz, Tuttlingen, Germany), while inspection of the nasal cavity was performed using rhinoscopy (Mini multi-purpose rigid telescope, 67030BA, Karl Storz). No remarkable findings were observed in the nasopharynx with retroflex rhinoscopy. A cystic tissue was identified in the left nasal cavity, occupying the entirety of the left nasal cavity and causing displacement of the right nasal cavity. The endoscopic examination revealed a cystic lesion with distinctive external and internal appearances. The external appearance of the cystic lesion demonstrated an elastic consistency of the cyst wall, which was notably vascularized, and its semi-transparent nature allowed visualization of the internal contents (Figure 3). Following artificial puncture of the cystic wall using a flexible 0.7 mm myringotomy needle (67071 XS, Karl Storz), flushing was performed through the cystic, translucent wall, followed by drainage. The internal contents included floating cholesterol particles. The ventral and caudal internal walls of the cyst exhibited a low proportion of vascularized mucosa, with these areas predominantly composed of smooth bone or cartilaginous tissue. A patchy lesion was found on the cyst floor, likely the source of cholesterol particles, suggesting a possible pathological origin (Figure 3). The cyst was aspirated endoscopically, and yellow serous exudate was obtained. Bacterial culture of exudate and nasal swabs from both cavities yielded negative results. Additionally, cystic tissue was obtained endoscopically for histopathological examination. The dog was well recovered from anesthesia.

**Figure 4:**
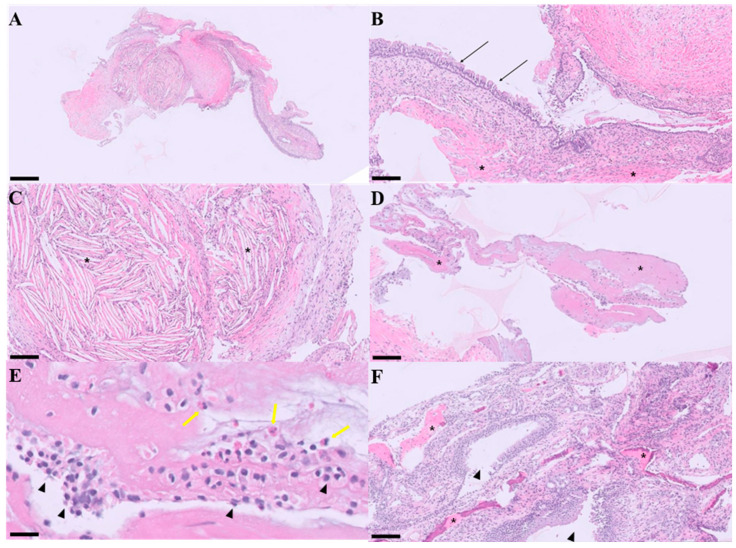
Microscopic Images of COREAH (Chondro-Osseous Respiratory Epithelial Adenomatoid Hyperplasia) Stained for Pathohistological Examination. The fragment is composed of fibrovascular tissue covered by well-differentiated ciliated pseudostratified to stratified squamous epithelium. H&E stain; scale bar, 500 μm (**A**). The surface is covered by pseudo-stratified ciliated epithelium (black arrows), and fibroepithelial proliferation (asterisks) is observed. H&E stain; scale bar, 100 μm (**B**). The stroma exhibits transparent, needle-shaped cholesterol crystals (asterisks), indicating cyst rupture or suggestive of cyst rupture and/or prior hemorrhagic degradation. H&E stain; scale bar, 100 μm (**C**). Osseous fragments are observed (asterisks). H&E stain; scale bar, 100 μm (**D**). Lymphoplasmacytic (arrowheads) and eosinophilic inflammation (yellow arrows) are present, suggesting a concurrent hypersensitivity reaction. H&E stain; scale bar, 20 μm (**E**). Proliferation of fibrovascular tissue is observed along with bone (asterisks), and glandular hyperplasia is also noted (arrowheads). Additionally, variable edema is present. H&E stain; scale bar, 100 μm (**F**).

On histopathologic examination, the mass was covered with columnar ciliated epithelium, and proliferation of well-differentiated respiratory epithelium and fibrovascular tissue was observed. Based on the anatomical location and diagnostic imaging findings, this lesion is considered a form of nasal hamartoma. The presence of bone fragments in some sections suggests that COREAH is present. COREAH can develop into an expansile paranasal sinus mass, and, as in this case, localized bone resorption may occur. Additionally, lymphoplasmacytic and moderate eosinophilic inflammation were observed, which may suggest a hypersensitivity reaction, such as an allergy. Furthermore, cholesterol crystals were surrounded by localized granulomatous inflammation, with variable edema also noted (Figure 4).

The patient was discharged with antibiotics prescription, gastrointestinal protectants, and hemostatic agents. Two weeks later, the owner reported no nasal discharge or congestion. However, nasal discharge, obstruction and stertor of the left nasal cavity recurred afterward, and the mass was subsequently removed through surgical excision rather than endoscopy. During the one-year follow-up period, no recurrence of clinical signs or evidence of COREAH was observed.

## 3. Discussion

COREAH is a rare benign growth characterized by the proliferation of respiratory epithelium alongside fibrovascular stroma and osseous fragments. In human medicine, it was first documented in the mid-1990s, with symptoms including nasal obstruction, discharge, and occasional headaches [1,2]. In contrast, canine cases are often presented with unilateral nasal discharge, stertor and soft tissue swelling associated with localized bone resorption [3,6].

In this case, the mass exhibited a translucent external wall, allowing for clear visualization of internal structures—an important feature observed during the endoscopic examination. This characteristic is particularly valuable for aiding in differential diagnosis and determining the nature of the internal structure.

Computed tomography (CT) scans further supported the diagnosis by showing a hypo-attenuated mass with localized bone resorption, a feature consistent with previously reported COREAH cases. Additionally, otitis media was observed on the CT scan, which may indicate a secondary inflammatory process in this dog. This finding has been documented in previous cases where sinonasal masses caused adjacent inflammatory conditions [8,9]. Similar inflammatory changes have been reported in other sinonasal lesions, including inflammatory polyps, mucoceles, osteomas, and even malignant tumors such as adenocarcinoma or squamous cell carcinoma, where local pressure or tissue invasion induces secondary otitis or rhinosinusitis. However, the cystic and translucent nature of the lesion, the presence of osseous fragments within respiratory epithelium, and the absence of invasive bone destruction collectively supported the diagnosis of COREAH rather than these alternative pathologies [10,11,12,13]. However, the absence of mineralization in this case contrasts with earlier findings, suggesting that COREAH may present with more variable radiological features than previously understood [3,5]. The extensive soft tissue involvement observed in this dog is rarely documented in veterinary cases, distinguishing it from earlier examples [5,14]. The significant soft tissue swelling initially raised concerns about more aggressive condition. It will be necessary to obtain CT images for evaluating the full extent of sinonasal masses and differentiating benign lesions like COREAH from malignant tumors [7,15]. 

Endoscopy played a critical role in diagnosing this case. The procedure provided a detailed view of the lesion, revealing floating yellowish, flaky osseous fragments within a cystic structure. This visualization, along with the mass’s translucent external wall, was crucial for differentiating COREAH from other sinonasal masses. The ability to obtain a minimally invasive biopsy through endoscopy further enabled histopathological evaluation consistent with COREAH [1,4]. The visibility of cystic contents, including flaky osseous fragments, is an important observation, as it suggests the lesion’s potential relationship with adjacent tissues, such as bone or cartilaginous structures [4]. This highlights the value of endoscopic evaluation in identifying sinonasal masses, particularly when imaging alone may initially suggest a more aggressive neoplastic process, as was suspected in this case [3].

This case report emphasizes endoscopic visualization and management of COREAH, distinguishing it from previous reports based mainly on CT imaging. Furthermore, unlike previous cases, the current case did not exhibit a mineralized soft tissue lesion, which serves as a distinguishing feature. The endoscopic approach provided real-time visualization, enabling precise tissue sampling and guiding surgical intervention, demonstrating a more comprehensive diagnostic strategy that can improve veterinary outcomes.

Histopathological examination revealed findings compatible with COREAH, including proliferation of respiratory epithelium, fibrovascular stroma, and the presence of osseous fragments. Although cartilaginous elements were not clearly identified, the coexistence of respiratory epithelial proliferation, fibrovascular stroma, and osseous components supports the diagnosis of COREAH, in reference to previous reports [3,14]. Additionally, eosinophilic and lymphoplasmacytic inflammation suggested an underlying hypersensitivity reaction, potentially an allergic response [5]. The presence of cholesterol crystals surrounded by granulomatous inflammation, likely resulting from chronic irritation or a prior cyst rupture, added a distinctive aspect to this case [14].

It is also possible that the left-sided COREAH was compressing the right side of the nasal cavity, leading to clinical symptoms such as nasal obstruction. Cases where unilateral sinonasal masses caused contralateral symptoms to have been reported in both veterinary and human literature, supporting this hypothesis [16,17]. The recurrence of nasal congestion raises questions about whether its origin is congenital or acquired. Congenital malformations, such as stenotic nares, are known to contribute to upper airway obstruction [18]. However, the improvement of symptoms following medical treatment suggests that inflammation played a role in the temporary resolution of clinical signs [17]. In this dog, clinical recurrence was observed after the initial endoscopic procedure, suggesting, like previously reported cases, that although inflammation may temporarily subside, the underlying cause, such as the mass, can persist and lead to recurrence [3]. Furthermore, CT findings of rhinitis in both nasal cavities suggest that chronic rhinitis may also be a contributing factor to the recurrence of symptoms [19]. However, after complete surgical excision of the mass, the patient remained free of clinical signs and showed no evidence of recurrence during the one-year follow-up period.

To the best of our knowledge, this is the first reported canine case of COREAH identified through endoscopic visualization, supported by CT imaging and histopathological examination. The main limitations of this study include the absence of cartilaginous elements on histopathology and the inability to perform additional immunohistochemical staining, such as cytokeratin and vimentin, due to the limited amount of residual tissue. Nevertheless, considering previous literature and the presence of characteristic histologic features, such as respiratory epithelial proliferation, fibrovascular stroma, and osseous trabeculae, this case can be confidently diagnosed as COREAH. This study provides novel insights that may enhance recognition and inform clinical decision-making in similar future cases.

## 4. Conclusions

This case report highlights that the combined use of CT, endoscopic, and histopathological evaluations offers complementary diagnostic value, providing a coherent basis for identifying COREAH as the underlying lesion. These findings underscore the importance of a multimodal diagnostic approach for accurate differentiation and effective management of sinonasal masses in dogs.

## Data Availability

The data that supports the findings of this case report are available from the corresponding author upon reasonable request.

## Abbreviations

The following abbreviations are used in this manuscript:

COREAH	Chondro-Osseous Respiratory Epithelial Adenomatoid Hamartoma
REAH	Respiratory Epithelial Adenomatoid Hamartoma
CT	Computed Tomography
